# Distribution of circulating tumor DNA in lung cancer: analysis of the primary lung and bone marrow along with the pulmonary venous and peripheral blood

**DOI:** 10.18632/oncotarget.19538

**Published:** 2017-07-25

**Authors:** Taichiro Goto, Yosuke Hirotsu, Kenji Amemiya, Takahiro Nakagomi, Daichi Shikata, Yujiro Yokoyama, Kenichiro Okimoto, Toshio Oyama, Hitoshi Mochizuki, Masao Omata

**Affiliations:** ^1^ Lung Cancer and Respiratory Disease Center, Yamanashi Central Hospital, Yamanashi, Japan; ^2^ Genome Analysis Center, Yamanashi Central Hospital, Yamanashi, Japan; ^3^ Department of Pathology, Yamanashi Central Hospital, Yamanashi, Japan; ^4^ University of Tokyo, Tokyo, Japan

**Keywords:** circulating tumor DNA, distribution, lung cancer, next-generation sequencing, plasma

## Abstract

Circulating tumor DNA (ctDNA), extracted from plasma, is a non-invasive surrogate biomarker. However, the distribution of ctDNA in the body still remains to be elucidated. In this study, resected lung tumors, with simultaneous blood and bone marrow samples, were analyzed to elucidate the distribution of ctDNA. Rib bone marrow, pulmonary venous blood (Pul.V) and peripheral blood (Peri.B) were obtained from 30 patients. The liquid samples were divided into cell pellets and supernatant by centrifugation; a total of 212 DNA samples were subjected to massively parallel sequencing. ctDNA was detected in 5 patients. Given that the frequency of mutations in the primary tumor was considered to be 100%, those in the other specimens were as follows; Pul.V plasma 20%, Peri.B plasma 11%, and the other samples 0%. Furthermore, ctDNA reflected the predominant mutations in the primary lesion. Clinically, the presence of ctDNA was associated with significantly poorer survival. These results suggest ctDNA “spill over” into an immediate outflow tract (Pul.V), and from there is disseminated to the entire body. Thus, it can be inferred that ctDNA reflects the cancer progression and could function as a prognostic marker.

## INTRODUCTION

Cancer may be described as a disease of genetic and epigenetic mutations [1]. Recent genome sequencing studies have revealed the genetic landscape of lung cancers and presented opportunities to identify specific biomarkers [2–4]. Circulating cell-free DNA in plasma or other body fluids has potentially transformative applications as a new-generation and non-invasive surrogate biomarker in cancer diagnosis and treatment [1, 5–10].

Serum-based protein biomarkers, such as the carcinoembryonic antigen and squamous cell carcinoma (SCC) antigen, are commonly used in the clinical setting; however, these markers are also produced by normal cells to a certain extent and thus do not always reflect cancer progression in all patients with lung cancer [11–13]. In contrast, cell-free DNA containing the tumor-specific variants, also known as circulating tumor DNA (ctDNA), is exclusively released by cancer cells, and thus is suggested to be a more specific and informative biomarker [1].

ctDNA is composed of small nucleic acid fragments and is released from tumor cells into the blood [14]. This release of ctDNA takes place through a variety of physiological cellular events, such as apoptosis, necrosis, and secretory release [10]. ctDNA is detectable in the plasma and serum of patients with advanced cancer [15–21].

Therefore, one of the most immediate applications of ctDNA is what has been termed “liquid biopsy” [9, 10, 16, 22–24]. Sensitive methods for detecting tumor-derived mutations in plasma may find applications in early detection, screening, disease prognosis, monitoring tumor dynamics over time, and the detection of minimal residual disease or resistant mutations [23, 25–28]. Although ctDNA has been detected in the peripheral blood, the mechanism and rate of release, as well as its distribution and natural course in the body, are still unknown.

In this study, we sought to verify the presence of ctDNA in operable primary lung cancer and to clarify its natural course and pathophysiology by determining its body distribution pattern. On the basis of previous studies on circulating tumor cells (CTCs) as well as disseminated tumor cells [29–35], we hypothesized that ctDNA exists in the pulmonary venous blood (Pul.V), peripheral blood (Peri.B), and/or bone marrow fluid (BM). Based on the hypothesis that ctDNA exists in these sites, even in relatively early lung cancers, we analyzed the distribution of ctDNA in the body. In addition, we analyzed the mutational profiles, pathohistology, and clinical characteristics of ctDNA-positive lung cancers, as well as the correlation between these findings and the postoperative survival rate. We also examined whether this minimal residual disease detection with ctDNA would be able to accurately identify patients at risk of cancer relapse.

## RESULTS

### The number of mutations found in the primary lesions

**Figure 1:**
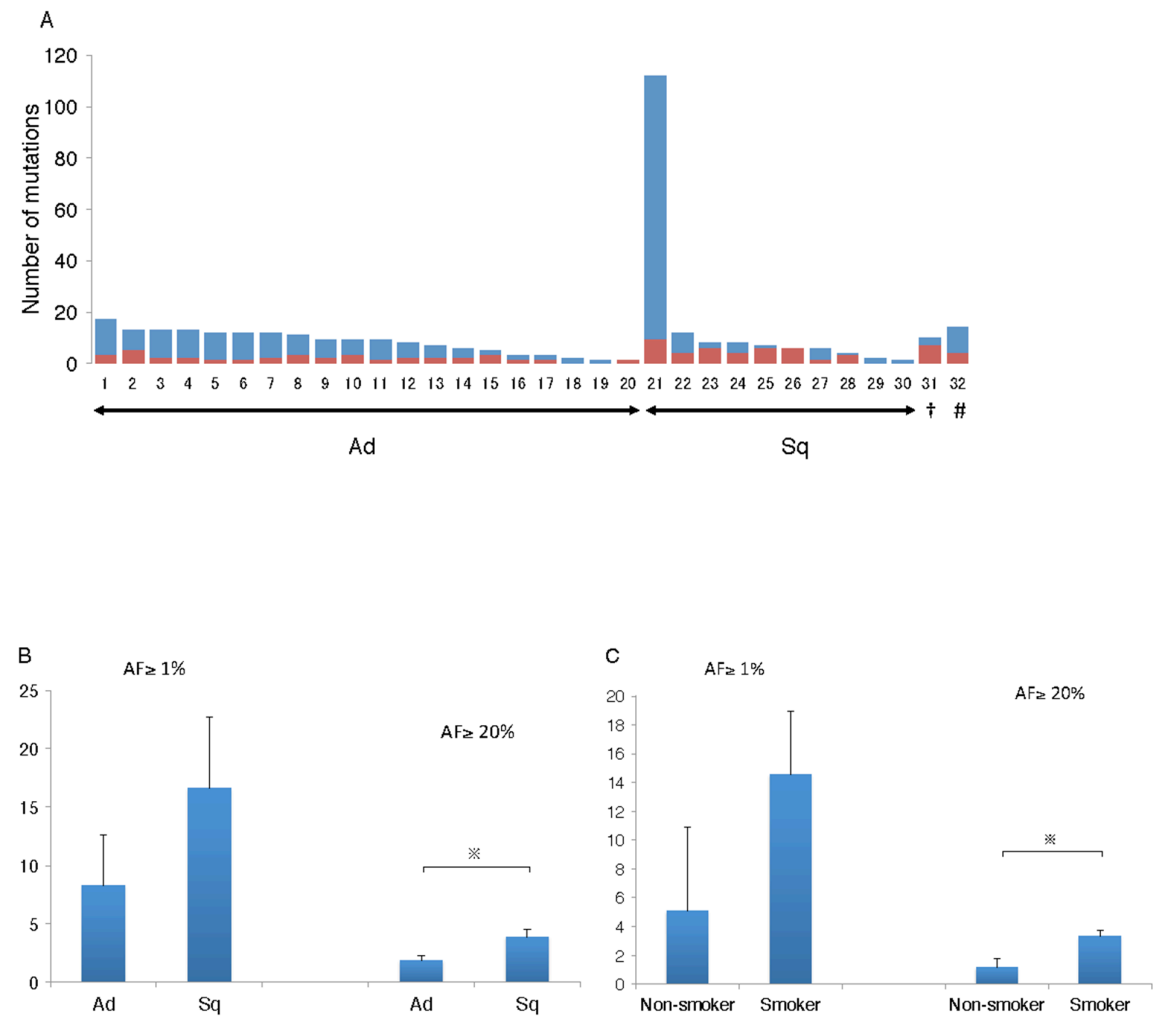
Number of mutations identified in the primary lesion **(A)** Deep sequencing of the samples revealed the number of mutations to be 11.1 ± 3.3 and 2.7 ± 0.4 with AF ≥ 1% and AF > 20%, respectively. Red bar indicates number of mutations with AF ≥ 20%. Blue bar indicates number of mutations with the limitation 20 > AF ≥ 1%. †, small cell carcinoma; #, large cell neuroendocrine carcinoma; Ad, adenocarcinoma; Sq, squamous cell carcinoma. **(B)** Squamous cell carcinoma contained significantly larger number of significant mutations with AF ≥ 20%, compared to adenocarcinoma. Ad, adenocarcinoma; Sq, squamous cell carcinoma; ※, *p* < 0.05. **(C)** Smokers had significantly larger number of significant mutations with AF ≥ 20% than non-smokers. ※, *p*<0.05.

Thirty-two primary tumors from 30 patients were analyzed by deep sequencing, which detected 11.1 ± 3.3 mutations with an allele fraction (AF) ≥ 1%, along with 2.7 ± 0.4 mutations with an AF ≥ 20% (Figure 1A). A comparison of the adenocarcinomas and the SCC histological types revealed no significant difference in the number of mutations with AF ≥ 1%. However, when only mutations with AF ≥ 20% were targeted, SCCs were found to harbor significantly more mutations than adenocarcinomas (Figure 1B). A comparison between smokers and non-smokers also showed that the number of mutations, especially significant mutations with AF ≥ 20%, was significantly larger among smokers (Figure 1C).

### Functional pathways of mutations found in lung cancers

**Figure 2:**
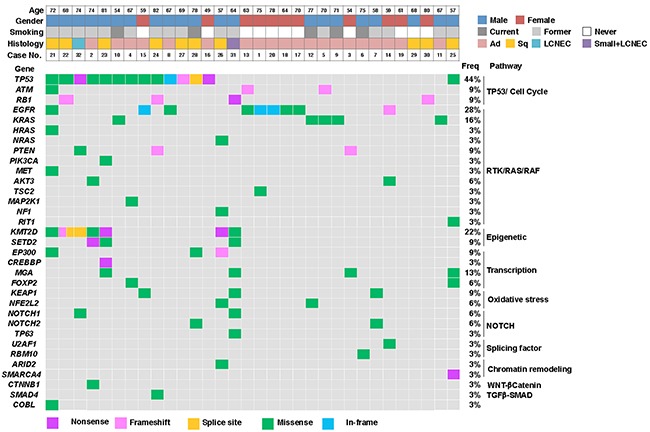
Lung cancer mutation profiles Most cancers had more than one mutation and involved several different functional pathways. Case No. indicates the number of the corresponding case in Figure 1A.

The significant mutations (AF ≥ 20%) that were detected in all 30 patients are shown in Figure 2 (Supplementary Tables 1 and 2). Although point mutations were most prevalent in general, frameshift and nonsense mutations were also frequently detected in tumor suppressor genes such as *RB1*, *PTEN*, and *TP53*. A comparison of the frequency of mutations in the functional pathways revealed that the RAS and TP53 pathways were affected in >50% of the cases, and that the frequency was significantly higher in these pathways than in the others (Supplementary Figure 1A). When the frequencies of the affected pathways were compared between adenocarcinomas and SCCs, the RAS pathway was found to be affected significantly more frequently in adenocarcinomas, while the chromatin remodeling, epigenetic, transcription, and TP53 pathways were affected significantly more frequently in SCCs (Supplementary Figure 1B). A comparison of cancers in smokers and non-smokers showed that the epigenetic pathway was affected significantly more frequently in lung cancers in smokers than non-smokers (Supplementary Figure 1C).

### Frequency of the ctDNA mutations in the different sampling sites

**Figure 3:**
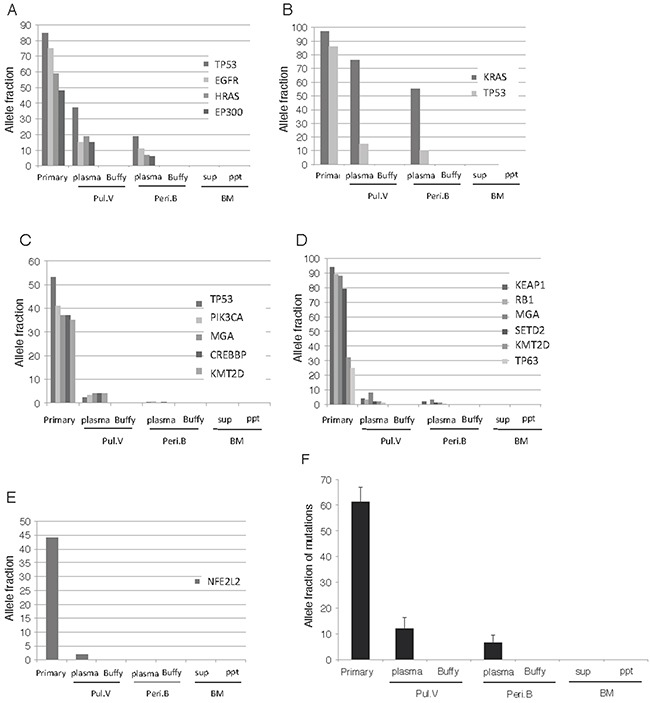
Analysis of the ctDNA distribution pattern **(A-E)** In 5 of the 30 patients, ctDNA was detected in the Pul.V and Peri.B plasma. Tumor DNA was not detected in the cell fractions or BM fluid, but was detected in three types of samples; the primary lesion, Pul.V plasma, and Peri.B plasma. Among these three samples, the gradients of AF for each mutation were always concordant, as follows. Primary lesion > Pul.V plasma > Peri.B plasma. Panels A–E correspond to Cases 1–5 in Table 1. **(F)** Compiling the data of these 5 patients, the allele fraction in the primary lesion was found to be significantly higher than that in the other 6 specimens. ※, *p* < 0.05. Assuming the primary tumor to be 100%, the frequencies of the mutations were as follows: Pul.V plasma, 20%; Peri.B plasma, 11%; Pul.V pellet, 0%; Peri.B pellet, 0%; supernatant of BM, 0%; and pellet of BM, 0%.

Using the mutations detected in the primary lesion as markers, we sought to detect identical mutations in the blood and BM fluid samples. In five of the 30 patients, identical mutations were detected in the ctDNA of Pul.V, and Peri.B plasma, and individual tumors released more than one type of ctDNA mutation (Table 1 and Figure 3A-3E). Identical mutations were not detected in the cell fractions or BM fluid. The AFs of the identical mutations detected in these five cases were compared among the primary lesion, Pul.V plasma, Peri.B plasma, supernatant of the BM suspensions, and cell fractions. Tumor DNA mutations were detected in three of these, the primary lesion, the Pul.V plasma, and the Peri.B plasma. The AF gradient for all the mutations was highest in the samples from the primary lesion, followed by that for mutations in the Pul.V plasma and then, the Peri.B plasma. Eighteen and 11 mutants were detected in the Pul.V plasma and Peri.B plasma samples, respectively (Table 1). Furthermore, of the 18 mutants detected in the Pul.V plasma samples, seven (38.9%) were not detected in the Peri.B plasma samples. Considering the primary tumor as 100%, the frequencies of the mutations were as follows: Pul.V plasma, 20%; Peri.B plasma, 11%; Pul.V pellet, 0%; Peri.B pellet, 0%; BM supernatant, 0%; and BM pellet, 0% (Figure 3F).

**Table 1:** Allele fractions of circulating tumor DNA detected in the samples

Case	Case No. in Supplementary Table 1	Histology	Gene	Primary lesion	Pul.V Plasma	Pul.V Buffy	Peri.B Plasma	Peri.B Buffy	BM.sup	BM.ppt
			TP53	85.0%	37.0%	-	19.0%	-	-	-
			EGFR	75.0%	15.0%	-	11.0%	-	-	-
			HRAS	59.0%	19.0%	-	7.0%	-	-	-
			EP300	48.0%	15.0%	-	6.0%	-	-	-
1	27	Sq	COBL	34.0%	-	-	-	-	-	-
			ATM	25.0%	-	-	-	-	-	-
			EP300	25.0%	-	-	-	-	-	-
			KMT2D	22.0%	-	-	-	-	-	-
			MET	21.0%	-	-	-	-	-	-
2	19	Ad	KRAS	97.0%	76.0%	-	55.0%	-	-	-
			TP53	86.1%	15.0%	-	10.0%	-	-	-
			SETD2	61.1%	-	-	-	-	-	-
			TP53	53.1%	2.3%	-	0.4%	-	-	-
3	21	Sq	PIK3CA	41.5%	3.3%	-	-	-	-	-
			MGA	37.3%	3.9%	-	-	-	-	-
			CREBBP	37.0%	4.1%	-	-	-	-	-
			KMT2D	34.9%	4.0%	-	-	-	-	-
			KEAP1	93.8%	3.7%	-	1.9%	-	-	-
			RB1	89.1%	3.0%	-	-	-	-	-
			MGA	87.8%	7.5%	-	2.8%	-	-	-
4	29	Small	SETD2	78.5%	1.6%	-	1.0%	-	-	-
			KMT2D	32.1%	2.0%	-	0.8%	-	-	-
			TP63	25.5%	0.9%	-	-	-	-	-
			NOTCH1	22.3%	-	-	-	-	-	-
			NF1	50.3%	-	-	-	-	-	-
			NRAS	48.6%	-	-	-	-	-	-
5	24	Sq	ARID2	45.0%	-	-	-	-	-	-
			NFE2L2	43.7%	2.0%	-	-	-	-	-
			EP300	40.9%	-	-	-	-	-	-
			KMT2D	29.1%	-	-	-	-	-	-

### Correlation between the frequency of tumor-derived DNA in the primary lesion and that in the ctDNA

**Figure 4:**
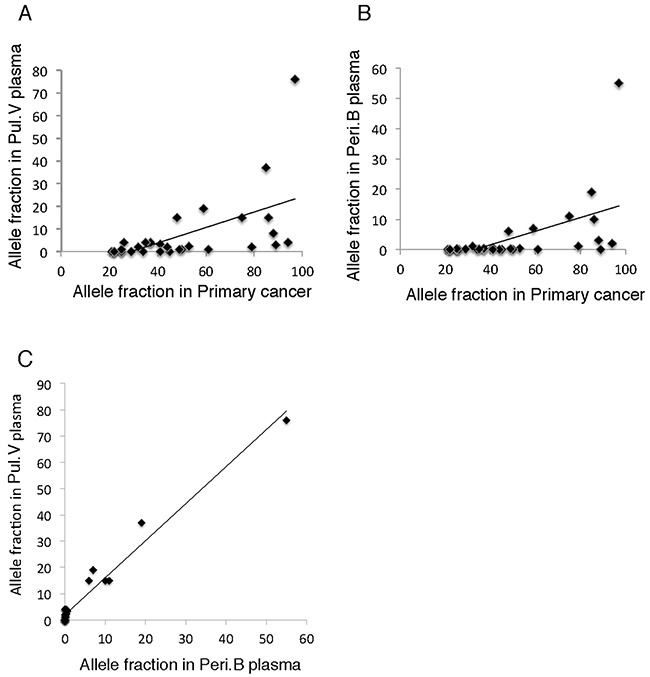
Correlation between allele fractions of the mutated genes in different samples **(A-B)** AF of the mutations detected in the primary lesion correlated significantly with that of mutations in the ctDNAs detected in Pul.V and Peri.B plasma (R = 0.57, *p* < 0.05 and R = 0.54, *p* < 0.05, respectively). **(C)** There was a clear correlation between the AFs of mutations in the ctDNA detected in Pul.V and Peri.B plasma (R = 0.98, *p* < 0.05).

We  examined the correlation between the AF values of mutations in the primary lesion and plasma samples to investigate the etiology of tumor-derived DNA in the body. The AF of the mutations in the primary lesion correlated significantly with that of the mutations in the ctDNA detected in the Pul.V and Peri.B plasma (R = 0.57, *p* < 0.05 and R = 0.54, *p* < 0.05, respectively) (Figure 4A and 4B). This suggested that ctDNA was more likely to be released into the blood when the mutations in the primary lesions had a higher AF value. A significant correlation was observed between the AF values of the mutations in the ctDNA detected in the Pul.V and Peri.B plasma (R = 0.98, *p* < 0.05). The AF of mutations in the ctDNA in the Pul.V plasma was higher than that in the Peri.B plasma. The approximation formula was *Y* = 1.42*X* + 1.81 (*X*: AF of the ctDNA in the Peri.B plasma; *Y*: AF of the ctDNA in the Pul.V plasma) (Figure 4C).

### Comparison of the number of mutations and functional pathways in cancers with and without ctDNA release

The number of mutations in lung cancers that released ctDNA into the plasma was compared with that in lung cancers that did not. With an AF threshold of either 1% or 20%, the number of mutations was significantly larger in lung cancers that released ctDNA (Table 2).

**Table 2:** The number of mutations in the cancers with or without circulating tumor DNA release

	ctDNA(-)	ctDNA(+)	*p* value
	(n = 27)	(n = 5)	
AF > 1%	7.8 ± 3.6	28.7 ± 8.1	<0.05
AF > 20%	2.0 ± 0.3	6.0 ± 0.7	<0.05

The epigenetic and transcription pathways were affected significantly more frequently in cancers that released ctDNA into the plasma than in those that did not (Supplementary Figure 2, *p* < 0.05).

### Number of mutations in the specimens, including the lung cancer-nonspecific ones

**Figure 5:**
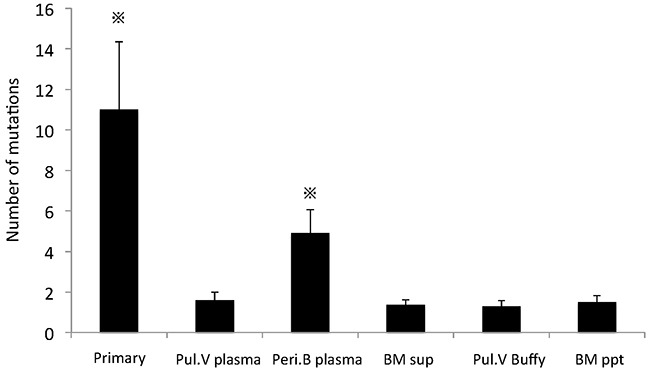
Number of mutations in the specimens, including lung cancer-nonspecific ones The number of mutations in the primary lesion and Peri.B plasma was significantly larger than that in the other 4 specimens. ※, *p* < 0.05. In this analysis, the number of mutations in Peri.B Buffy was set at 0, because these leukocytes were utilized as the normal controls.

In comparison with the total number of cell-free mutations with AF ≥ 1% that were detected in the samples, including mutations that did not correspond to those detected in the primary lung cancer lesion, the number of mutations was significantly larger in the samples obtained from the primary lesion and Peri.B plasma than in those obtained from the other 4 samples (Figure 5, *p* < 0.05; Supplementary Table 3). In particular, the number of mutations detected in Peri.B plasma was significantly larger than that in Pul.V plasma (Figure 5, *p* < 0.05).

### Correlations between clinical conditions and ctDNA

The clinical aspects of lung cancer were compared between ctDNA-positive and ctDNA-negative lung cancers (Table 3 and Supplementary Table 1). It was found that men and smokers were significantly more likely to be positive for ctDNA (Table 3, *p* < 0.05). Moreover, the tumor size was significantly larger and, histologically, the proportion of SCCs was significantly higher for ctDNA-positive lung cancers (Table 3, *p* < 0.05). Patients with ctDNA-positive lung cancer were found to be more likely to have advanced-stage disease and to have undergone non-curative surgery (Table 3, *p* = 0.08 and *p* = 0.09, respectively). Pathological vessel and lymphatic invasions were not associated with the presence of ctDNA. Through a multivariate analysis, we identified size, histology, and pathological stage of the cancer as factors that affect ctDNA release (*p* < 0.05); no correlations were observed for patient age, gender, and smoking habit.

**Table 3:** Clinical and pathological characteristics of patients with or without detected circulating tumor DNA

	ctDNA(-)	ctDNA(+)	*p* value
Number of patients	25	5	-
Male/Female	14/11	5/0	<0.05
Age	68.4 ± 1.8	65.6 ± 4.0	NS
Smoking habit			
smoker/non-smoker	15/10	5/0	<0.05
Tumor size (mm)	30.8 ± 4.7	64.0 ± 10.6	<0.05
Surgical curativity			
curative/non-curative	23/2	3/2	0.09
Histology			
Ad/ Sq/ others	18/6/1	1/3/1	<0.05
Pathological stage			
I/II/III/IV	17/3/3/2	1/1/1/2	0.08
Pathological vessel invasion			
present/absent	9/16	3/2	NS
Pathological lymphatic invasion			
present/absent	7/18	1/4	NS
Adjuvant chemotherapy			
performed/ not performed	7/18	3/2	NS

Ad: adenocarcinoma, Sq: squamous cell carcinoma, NS: not significant.

### Survival rate of lung cancer patients with or without plasma ctDNA

**Figure 6:**
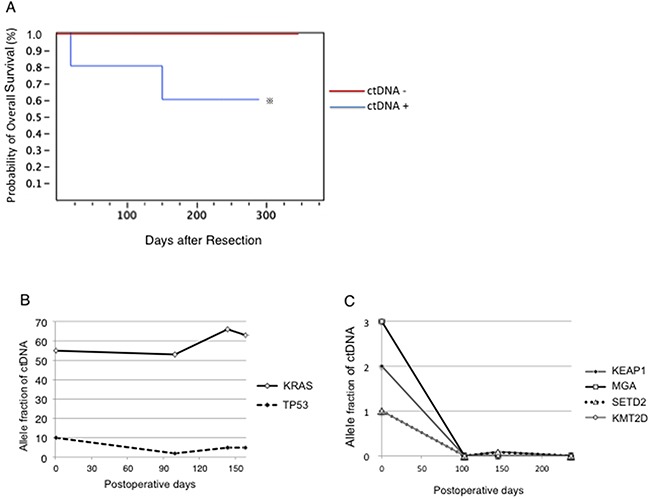
Clinical outcomes of the presence of ctDNA in the plasma **(A)** Five patients with plasma ctDNA exhibited significantly poorer survival than the 25 patients without ctDNA in their plasma. ※, *p* < 0.05. **(B-C)** Changes in ctDNA after surgery reflects cancer progression and predicts the outcome. Panels B and C represent Cases 2 and 4, respectively, in Table 1.

The five patients with plasma ctDNA had significantly poorer outcomes than 25 patients without ctDNA in their plasma (Figure 6A). Two patients who died of cancer had mutated plasma ctDNA with extremely high AF (Table 1 and Supplementary Table 1).

### Postoperative changes in the plasma ctDNA

The ctDNA levels in the Peri.B plasma were monitored after surgery in two patients with Peri.B plasma ctDNA (Figure 6B and 6C). The patient with adenocarcinoma was pathologically classified as T2aN0M1a at stage IV (Figure 6B). Platinum-based systemic chemotherapy was performed continuously; however, the ctDNA levels remained high after surgery, and the patient died on day 158 after the operation. The other patient had small cell carcinoma clinically classified as T2aN0M1a at stage IV (Figure 6C). Following surgery, platinum-based systemic chemotherapy was performed. The ctDNA levels were undetectable after surgery and chemotherapy, and the patient is still alive without relapse, as of postoperative day 470.

## DISCUSSION

In this study, we found that the ctDNA frequency in the sampling sites, assuming that it was 100% in the primary tumor, was as follows: Pul.V plasma, 20%; Peri.B plasma, 11%; Pul.V pellet, 0%; Peri.B pellet, 0%; BM supernatant, 0%; and BM pellet, 0%. Furthermore, it was revealed that cancer-specific mutations were more frequent in Pul.V than in Peri.B plasma. Thus, a“spill-over” of ctDNA into the immediate outflow tract (Pul.V) occurred; it was then diluted, but still present, in the returning circulation (Peri.B). ctDNA has been reported to have a short half-life (2 h) [23]; once it is spilled out of cancer cells, it disseminates and disappears rapidly. Consequently, it was assumed that ctDNA levels decreased below the detectable level in BM fluid and other body fluids in regions distant from tumors. The AF of the ctDNA correlated with that of the DNA detected in the primary lesion, suggesting that ctDNA might accurately reflect predominant mutations that arise in the primary lesion.

Individual lung cancers could harbor more than one mutation, contributing to intratumor heterogeneity [2–4, 36]. In the current study, the number of mutations was larger in SCCs than in adenocarcinomas, and larger in smokers than in non-smokers; these results were consistent with previously reported findings [37–39]. In SCCs and in smokers with lung cancer, it is assumed that the cancer is poorly differentiated because of the accumulation of a higher number of mutations than in non-SCCs and non-smokers, respectively. There have been reports of lower survival rates in SCCs than adenocarcinomas, even at the same disease stage [40, 41].

In clinical practice, there are no standard methods for determining the driver mutation among the several mutations found in lung cancer. In the current study, as described in many reports, we considered mutations with higher AF more likely to be driver mutations. Our data indicated that individual lung cancers harbor 2.7 significant mutations on average; selecting the mutation to be target for treatment among these will be a major issue for medical treatment in the future.

The RAS and TP53 pathways have been reported to be the major pathways involved in lung cancer [2–4, 37]; our results were consistent with these reports. Adenocarcinomas with EGFR mutations is prevalent among Asians [38, 42]. The current study revealed that whereas the RAS pathway was affected mainly in adenocarcinomas, the TP53 pathway was affected mostly in patients with SCCs, as reported in many studies [2, 37]. Generally, pathways such as those for chromatin remodeling, epigenetics, and transcription are thought to be relatively minor pathways in lung cancer; however, in SCCs, smoking presumably triggers the accumulation of mutations in such pathways through a progressive or evolutionary process, which results in conversion to a poorly differentiated phenotype. In fact, the tumors in none of the 30 patients in the current study exclusively harbored mutations of only one of these three pathways. It is assumed that SCCs arise and progress through the coordinated effects of these and other mutated pathways.

In five of the 30 patients with lung cancer, ctDNA was detected in plasma at a low detection rate (16.7%). In this study, peripheral blood was drawn before surgery, while pulmonary venous blood was drawn after surgery. Therefore, it is possible that the surgery itself caused ctDNA to be released into the blood. Despite this possibility, the strong correlations between the mutations in the pulmonary venous and peripheral blood, as shown in Figure 4C, suggested that they were derived from the same mechanism of release. From the clinical perspective, it is important to determine ctDNA-positive cases. The current study, which included 30 surgical patients selected in an unbiased fashion, is unique in that patients with various histological types at different disease stages were included. The clinical features associated with ctDNA detection were large cancer size, advanced stage, and SCC histology. In this context, tumor burden and tumor cell proliferation rates might have a substantial role in the physiology and rate of ctDNA release. In addition, we also analyzed the mutational profiles of ctDNA-positive cases. As shown in Table 2 and Supplementary Figure 2, ctDNA was detected more frequently in patients with cancers that harbored many mutations and/or cancers that harbored mutations of the epigenetic or transcription pathways. This should be interpreted as a consequence of ctDNA release from SCCs and/or large lung cancers.

In the current study, Pul.V, Peri.B, and BM fluid samples were used to perform deep sequencing on both cell and supernatant fractions. There are two accessible sources of tumor DNA in the blood: CTCs and ctDNA [43, 44]. Although a simple comparison of the two sources is difficult because of the differences in the detection methods used, the current study detected no mutated genes in the cell fractions, even in patients positive for ctDNA. It was evident that ctDNA was more readily detectable than CTCs. Many recent studies have reported that ctDNA levels were higher than the CTC levels [15, 45, 46], and our results were consistent with these reports. Furthermore, we had previously reported that ctDNA in plasma was a more sensitive and clinically useful biomarker than DNA in the BM fluid [47].

As shown in Figure 6A, there was a marked correlation between ctDNA and poor survival rate, suggesting that detecting ctDNA with targeted deep sequencing could provide useful prognostic information on patients after surgery. Furthermore, the two patients who died had the highest AF of ctDNA in the peripheral blood, while another three patients with AF in peripheral blood < 3% survived without recurrence, indicating that ctDNA-positivity and AF might be associated with poor survival.

ctDNA also showed a more dynamic range that correlated with changes in the tumor burden, as shown in Figure 6B. We demonstrated that noninvasively tracking mutations in plasma DNA could help detect residual diseases that standard surgery failed to eradicate, and thus identify patients with high risk of recurrence. In the current study, cancer-specific mutations were detected more frequently in Pul.V plasma, and the AF of ctDNA in the Pul.V plasma was approximately twice that in the Peri.B plasma. In fact, a number of mutated ctDNA fragments were detected only in the Pul.V plasma. Thus, collecting Pul.V blood from surgical samples instead of the conventional Peri.B sampling could further facilitate the detection of ctDNA, and consequently, enable a more accurate assessment of disease progression. However, ctDNA have not been confirmed to act as reliable markers of cancer progression. Our findings need to be validated by larger and comparative studies in the future.

Our chosen approach for collecting pulmonary venous blood could potentially be used in interventional radiology. In patients with lung cancer, pulmonary venous blood samples could be collected with a catheter for clinical use. Our results indicated that the ctDNA released from lung cancer into the pulmonary venous blood was more lung cancer-specific, abundant, and precise than the ctDNA in the peripheral blood. Thus, pulmonary venous blood appeared to be a better source of ctDNA than peripheral blood, in terms of both sensitivity and specificity. In the future, measuring ctDNA in the Pul.V plasma might be useful for monitoring tumor dynamics, detecting minimal residual diseases, detecting resistant mutations, and other diagnostic/prognostic procedures.

It has been reported that a higher burden of non-synonymous mutations in tumors is associated with clinical benefits of immune checkpoint therapy [48]. As shown in the current study, ctDNA is more likely to be released in lung cancers with a higher mutation burden; thus, measuring the amount of ctDNA might provide pivotal information during the course of immune checkpoint therapy. Although we showed that the liquid biopsy approach is promising, its clinical utility has not been evaluated in a cohort study. Our results could lead to various such cohort studies, including those of adjuvant chemotherapy after lung cancer surgery.

One limitation of the current study that should be considered is the small sample size. Nevertheless, the results have translational implications and suggest important avenues for future research.

We expect that ctDNA will be increasingly used as a tumor progression marker for assessing the therapeutic effects of anticancer agents and other processes in clinical practice. Evolutionary changes that take place within cancers could affect both the mutational spectrum of the disease and its sensitivity to treatment [22, 49–54]. We envision a time when blood-based gene profiling supplements tumor genotyping to track the heterogeneous and evolving genomic landscape, thus informing treatment strategies and realizing safer and more precise strategies for cancer management.

In conclusion, we showed that, in the natural course of the disease, ctDNA spills directly out of tumors into the pulmonary venous blood and is then disseminated to the entire body. As ctDNA reflects the predominant mutations in the primary lesion and bears promise as a prognostic marker that closely reflects disease progression, its measurement might be used in the future as a “liquid biopsy” in the real sense.

## MATERIALS AND METHODS

### Patients and sample preparation

We selected the first 30 patients with lung cancer admitted to our hospital between September 2014 and February 2015. Before surgery, we explained the surgical procedures to each patient, including the rib resection and sample collection from the lung, blood, and bone marrow. All the patients provided written informed consent for these procedures as well as for the genetic research, which was performed in accordance with protocols approved by the Institutional Review Board of our hospital.

A peripheral blood sample was drawn from the patients just prior to surgery. According to our policy, standard resection of lung cancer (i.e., lobectomy) was performed with an open-chest approach. Immediately after the lobectomy, the pulmonary vein was punctured in order to collect the pulmonary venous blood. Blood samples were collected in EDTA-2Na tubes. During surgery, specimens (approximately 1 cm in length) were collected by dissecting the 5^th^ and 6^th^ ribs. Immediately after rib resection, the bone marrow space in the rib was repeatedly washed until all the components were removed and the bone marrow cell suspensions thus were obtained. After collecting the blood and bone marrow samples, they were immediately sent to the genome analysis center of our hospital. Centrifugation was initiated within 1 h of acquisition of each sample. The buffy coat (pulmonary venous blood and peripheral blood) and cell pellets (rib bone marrow fluid) were isolated after centrifugation of these samples at 820 ×*g* for 10 min at 25°C. Plasma from the blood and supernatant of the bone marrow fluid was centrifuged at 20,000 ×*g* for 10 min and the supernatant was subsequently transferred to sterile tubes and stored at −80°C before DNA extraction. Total DNA was extracted from lymphocytes and cell pellets using the QIAamp DNA Blood Mini Kit (Qiagen, Tokyo, Japan), and DNA was purified from the plasma and supernatant using the QIAamp Circulating Nucleic Acid Kit (Qiagen). Serial sections of formalin-fixed, paraffin-embedded (FFPE) tissue were stained with hematoxylin-eosin and microdissected using an ArcturusXT laser-capture microdissection system (Thermo Fisher Scientific, Tokyo, Japan). Tumor DNA was extracted using the QIAamp DNA FFPE Tissue Kit (Qiagen).

### Assessment of DNA integrity with quantitative real time PCR

DNA fragmentation in the FFPE DNA was assessed using the TaqMan RNase P Detection Reagents Kit and the FFPE DNA QC Assay on a ViiA7 Real-Time PCR instrument (Thermo Fisher Scientific). Human control genomic DNA (included in the TaqMan RNase P Detection Reagents Kit) was serially diluted 4 times for a 5-point standard curve and the absolute DNA concentrations were determined. Assessment of DNA fragmentation was estimated with the ratio of the DNA (relative quantification, RQ) obtained for the long amplicon (256 bp) to the short amplicon (87 bp). The RQ value was used as an indicator of the degradation level of genomic DNA.

### Targeted deep sequencing and data analysis

A panel targeting the exons of 53 lung cancer-associated genes was established to perform targeted sequencing, as described in our previous study [47]. The genes were selected based on our literature review and the following criteria (Supplementary Table 4): (a) genes often involved in lung cancer, obtained from TCGA [2, 3] and other projects [55–59] and (b) genes frequently mutated in lung cancer, obtained from the COSMIC database (http://cancer.sanger.ac.uk/cancergenome/projects/cosmic). The primers for the targeted sequencing were designed using the Ion AmpliSeq designer software (Thermo Fisher Scientific), as reported in a previous study [60, 61]. Sequencing libraries were prepared using the Ion AmpliSeq Library kit (Thermo Fisher Scientific), according to the manufacturer's instructions. After barcode ligation using the Ion Xpress Barcode Adapters kit (Thermo Fisher Scientific), library samples were purified using the Agencourt AMPure XP reagent (Beckman Coulter, Tokyo, Japan) and then quantified using the Ion Library Quantitation Kit (Thermo Fisher Scientific). The libraries were templated with the Ion PI Template OT2 200 Kit v3 (Thermo Fisher Scientific). Sequencing was carried out on an Ion Proton (Ion Torrent, Thermo Fisher Scientific) using the Ion PI Sequencing 200 Kit v3. The sequence data were processed using the standard Ion Torrent Suite Software running on the Torrent Server. Raw signal data were analyzed using Torrent Suite version 4.0. The pipeline included signaling, processing, base calling, quality score assignment, and read alignment with the human genome 19 reference (hg19), along with quality control of mapping and coverage analysis. After data analysis, the single nucleotide variants, insertions, and deletions were annotated using the Ion Reporter Server System (Thermo Fisher Scientific); lymphocytes from peripheral blood DNA were used as controls to detect variants (Tumor-Normal pair analysis). The average sequencing coverage depth and uniformity were 593-fold and 83%, respectively (Supplementary Table 5). To detect somatic mutations in tumors with confidence, mutations with a variant allele fraction ≥ 20% were used. These somatic mutations were sought in the blood and bone marrow as well, and sequence data were visually confirmed using the Integrative Genomics Viewer.

### Statistical analysis

Continuous variables were presented as the mean ± SE and compared using the unpaired Student's *t*-test. One-way analysis of variance and Tukey-Krammer multiple comparisons tests were used to detect statistical significance between groups. Chi-square tests were used to compare the categorical data between groups. Multivariate analyses were performed to identify factors that affected the release of ctDNA. Correlation between samples was calculated using the Pearson's correlation coefficient. Overall survival time was defined as the period from the day of operation to the day of death or the day of the last follow-up evaluation. Survival was assessed using the Kaplan-Meier method, and comparisons among the survival curves were performed using the log-rank test. All statistical analyses were performed using the JMP function in the SAS software package (SAS Institute, Inc., Cary, NC). *P* values less than 0.05 in two-tailed analyses were considered to denote statistical significance.

## SUPPLEMENTARY MATERIALS FIGURES AND TABLES











## Abbreviations

ctDNA: circulating tumor DNA
BM: bone marrow
Pul.V: pulmonary venous blood
Peri.B: peripheral blood
SCC: squamous cell carcinoma
CTC: circulating tumor cell
AF: allele fraction
FFPE: formalin fixed, paraffin embedded
