# Supplementary material for: Distribution of circulating tumor DNA in lung cancer: analysis of the primary lung and bone marrow along with the pulmonary venous and peripheral blood
Source: Oncotarget. 2017 Jul 25;8(35):59268–81. doi: 10.18632/oncotarget.19538 (PMC5601731; doi:10.18632/oncotarget.19538)
Supplement: Supplementary file 1 [file oncotarget-08-59268-s001.pdf]

Distribution of circulating tumor DNA in lung cancer: analysis of the primary lung and bone marrow along with the pulmonary venous and peripheral blood

SUPPLEMENTARY MATERIALS

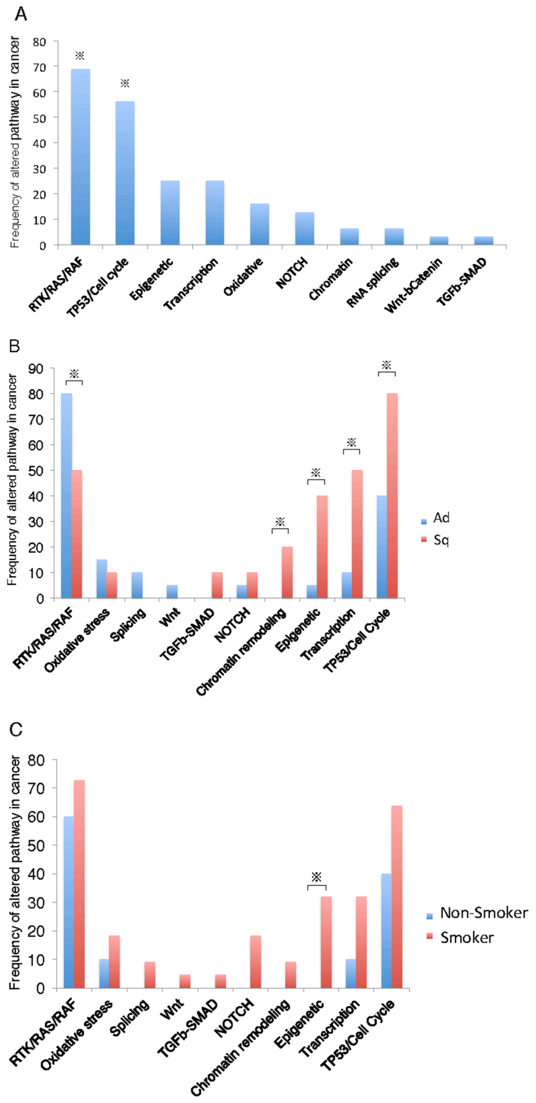

Supplementary Figure 1: Lung cancer mutation profiles in association with the functional pathways. (A) RAS and TP53 pathways are considered the major pathways in the oncogenesis of lung cancers. ※, $p < 0.05$, compared to the other 8 pathways. **(B)** RAS pathway is predominantly involved in adenocarcinoma, while chromatin remodeling, epigenetic, transcription, and the TP53 pathway are predominantly involved in squamous cell carcinoma. Ad: adenocarcinoma, Sq: squamous cell carcinoma. ※, $p < 0.05$. **(C)** Epigenetic pathway is affected significantly more frequently in lung cancer in smokers than non-smokers. ※, $p < 0.05$.

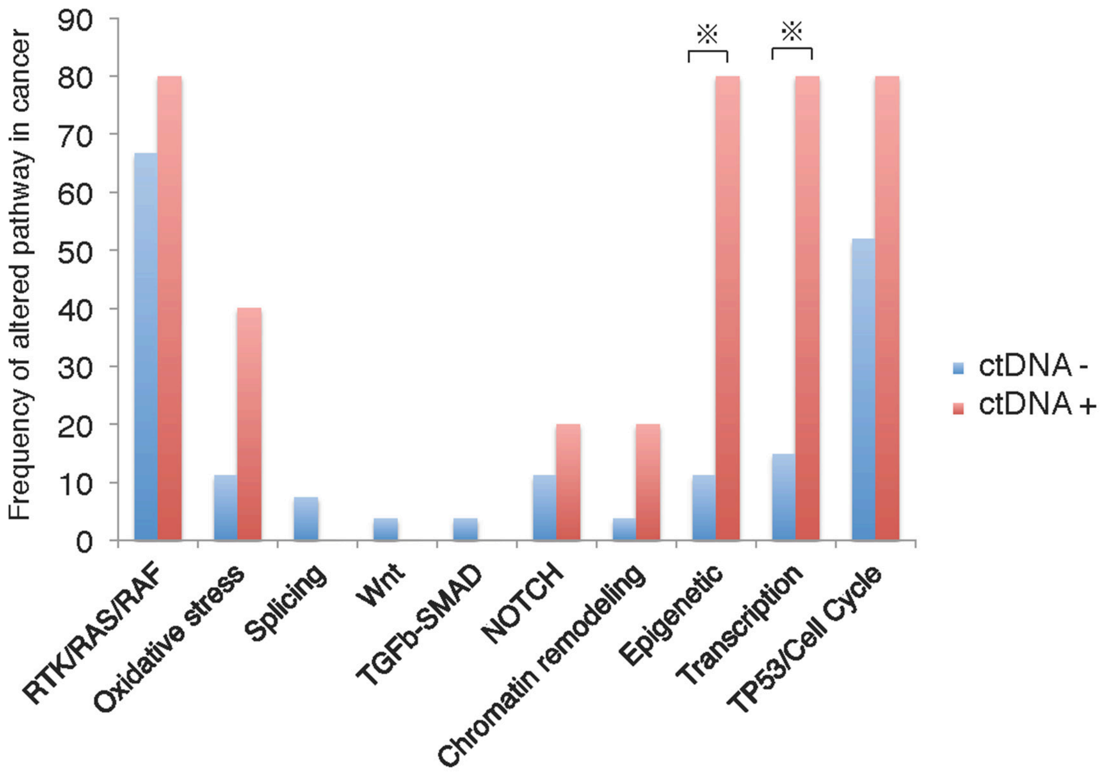

Supplementary Figure 2: Functional pathways of cancers with or without ctDNA release. The epigenetic and transcription pathways are involved significantly more frequently in cancers with ctDNA released into plasma than in those without ctDNA release. ※, $p < 0.05$, χ^2 test. Blue bar indicates cancers without ctDNA release (n = 27) and red bar indicates cancers with ctDNA release (n = 5).

Supplementary Table 1: Characteristics of the patients enrolled in the study

See Supplementary File 1

Supplementary Table 2: Mutations detected in the primary lung lesions

See Supplementary File 2

Supplementary Table 3: The number of cell-free somatic mutations containing ctDNA in analyzed samples

See Supplementary File 3

Supplementary Table 4: The genes targeted in the cancer

See Supplementary File 4

Supplementary Table 5: Sequencing read and coverage analysis

Sample	Mapped reads	On target	Mean depth	Uniformity
BM ppt	1852758	90.1%	607.9	85.6%
BM sup	2152505	90.2%	667.9	84.0%
Peri.B Buffy	2148937	96.0%	735.1	86.6%
Peri.B plasma	1544693	79.5%	414.0	75.8%
Pul.V Buffy	1871567	96.0%	632.0	86.3%
Pul.V plasma	1937329	60.3%	395.1	86.7%
Tumor	2214684	94.5%	699.0	73.3%

ppt: precipitant, sup: supernatant, Buffy: Buffy coat.
