# Supplementary material for: Distribution of circulating tumor DNA in lung cancer: analysis of the primary lung and bone marrow along with the pulmonary venous and peripheral blood
Source: Oncotarget. 2017 Jul 25;8(35):59268–81. doi: 10.18632/oncotarget.19538 (PMC5601731; doi:10.18632/oncotarget.19538)
Supplement: Supplementary file 2 [file oncotarget-08-59268-s002.docx]

Supplementary Table 1: Characteristics of the patients enrolled in the study

| **Case** | **Age** | **Gender** | **Smoking** | **B.I.** | **Symptoms** | **size (mm)** | **pTNM** | **p stage** | **histology** | **Curability** | **ctDNA** | **Adjuvant** | **Post ope** |
| --- | --- | --- | --- | --- | --- | --- | --- | --- | --- | --- | --- | --- | --- |
| 1 | 71 | male | current | 800 | -  -  - | 10 | 1a 0 0 | IA | invasive Ad, ly0, v0, pl0 | R0 | - | - | 15m, no rec |
| 2 | 78 | female | never | 0 | - | 10 | 1a 0 0 | IA | invasive Ad, ly0, v0, pl0 | R0 | - | - | 15m, no rec |
| 3 | 75 | male | current | 825 | - | 12 | 1a 0 0 | IA | MIA, ly0, v0, pl0 | R0 | - | - | 15m, no rec |
| 4 | 49 | female | never | 0 | - | 15 | 1a 0 0 | IA | invasive Ad, ly0, v0, pl0 | R0 | - | - | 14m, no rec |
| 5 | 75 | female | never | 0 | - | 20 | 1a 0 0 | IA | invasive Ad, ly0, v0, pl0 | R0 | - | - | 17m, no rec |
| 6 | 70 | male | former | 800 | - | 22 | 1b 0 0 | IA | invasive Ad, ly0, v0, pl0 | R0 | - | - | 18m, no rec |
| 7 | 64 | female | former | 80 | - | 25 | 1b 0 0 | IA | MIA, ly0, v0, pl0 | R0 | - | - | 15m, no rec |
| 8 | 63 | female | never | 0 | - | 25 | 1b 0 0 | IA | invasive Ad, ly0, v0, pl0 | R0 | - | - | 19m, no rec |
| 9 | 59 | female | never | 0 | - | 25 | 2a 0 0 | IB | invasive Ad, ly0, v1, pl1 | R0 | - | - | 16m, no rec |
| 10 | 67 | male | former | 800 | - | 32 | 2a 0 0 | IB | invasive Ad, ly0, v0, pl0 | R0 | - | - | 15m, no rec |
| 11 | 59 | female | former | 700 | - | 33 | 2a 0 0 | IB | invasive Ad, ly0, v0, pl0 | R0 | - | ＋ | 14m, no rec |
| 12 | 70 | female | never | 0 | - | 35 | 2a 0 0 | IB | invasive Ad, ly0, v0, pl0 | R0 | - | ＋ | 14m, no rec |
| 13 | 61 | female | never | 0 | - | 35 | 2a 0 0 | IB | invasive Ad, ly0, v0, pl0 | R0 | - | - | 16m, no rec |
| 14 | 77 | male | current | 1250 | - | 35 | 2a 0 0 | IB | invasive Ad, ly0, v1, pl0 | R0 | - | - | 18m, no rec |
| 15 | 74 | male | former | 600 | - | 50 | 2a 0 0 | IB | invasive Ad, ly0, v1, pl0 | R0 | - | - | 18m, no rec |
| 16 | 54 | female | never | 0 | - | 16 | 1a 2 0 | IIIA | invasive Ad, ly1, v0, pl0 | R0 | - | ＋ | 15m, rec |
| 17 | 58 | male | former | 350 | - | 28 | 1b 2 0 | IIIA | invasive Ad, ly1, v0, pl0 | R0 | - | ＋ | 15m, no rec |
| 18 | 67 | male | former | 1400 | - | 15 | 2a 0 1a | IV | i) invasive Ad, ly0, v0, pl1, ii) invasive Ad, ly0, v0, pl0 | R1 | - | ＋ | 17m, no rec |
| 19 | 54 | male | current | 600 | bil. leg edema | 50 | 2a 0 1a | IV | invasive Ad, ly0, v0, pl0 | R1 | ＋ | ＋ | 5m, dead |
| 20 | 68 | male | former | 1800 | - | 20 | 1a 0 0 | IA | Sq, ly1, v1, pl0 | R0 | - | - | 17m, no rec |
| 21 | 81 | male | former | 400 | - | 35 | 2a 0 0 O | IB | Sq, ly0, v1, pl1 | R0 | ＋ | - | 16m, no rec |
| 22 | 82 | male | former | 1100 | - | 60 | 2b 0 0 | IIA | Sq, ly0, v0, pl0 | R0 | - | - | 16m, no rec |
| 23 | 69 | male | former | 900 | - | 35 | 3 0 0 | IIB | Sq, ly1, v1, pl1 | R0 | - | ＋ | 14m, no rec |
| 24 | 57 | male | former | 600 | - | 60 | 2b 1 0 | IIB | i) Sq, ly1, v1, pl1, ii) Sq, ly0, v1, pl2 | R0 | ＋ | ＋ | 15m, no rec |
| 25 | 80 | female | never | 0 | - | 90 | 3 0 0 | IIB | Sq, ly2, v2, pl3 | R0 | - | - | 14m, rec |
| 26 | 78 | male | current | 900 | - | 60 | 2b 2 0 | IIIA | Sq, ly1, v1, pl0 | R0 | - | - | 14m, no rec |
| 27 | 72 | male | former | 1500 | hyponatremia | 140 | 3 1 0 | IIIA | Sq, ly0, v1, pl1 | R0 | ＋ | - | 1m, dead |
| 28 | 68 | male | never | 0 | - | 35 | 2a 2 1a | IV | Sq, ly0, v1, pl1 | R1 | - | ＋ | 16m, no rec |
| 29 | 64 | male | former | 1200 | - | 35 | 2a 0 1a | IV | small, ly0, v0, pl2 | R1 | ＋ | ＋ | 14m, no rec |
| 30 | 74 | male | former | 1200 | - | 28 | 1b 0 0 | IA | LCNEC, ly1, v1, pl0 | R0 | - | - | 18m, no rec |
