# Supplementary material for: Distribution of circulating tumor DNA in lung cancer: analysis of the primary lung and bone marrow along with the pulmonary venous and peripheral blood
Source: Oncotarget. 2017 Jul 25;8(35):59268–81. doi: 10.18632/oncotarget.19538 (PMC5601731; doi:10.18632/oncotarget.19538)
Supplement: Supplementary file 3 [file oncotarget-08-59268-s003.docx]

Supplementary Table 2: Mutations detected in the primary lung lesions

**Tumor Var.**

**Case**

**Histology**

**Gene**

**Protein**

**Position**

**Ref.**

**AF**

1

Ad

KRAS

p.Gln61His

chr12:25380274

T GAATTAAGAGA

G

39%

2

Ad

EGFR

p.E746-S752 delinsV

chr7:55242466

GTT

29%

AGCAACATCT

3

4

Ad Ad

RBM10 TP53

p.Asn371Ile p.Arg306Ter

chrX:47039294 chr17:7577022

A G

T A

41%

44%

EGFR EGFR TSC2

p.Glu746_Arg748del p.Ala750Pro p.Cys519Ser

chr7:55242465 chr7:55242478 chr16:2114384

GGAATTAAGA G

T

G C A

51%

50%

21%

5

Ad

6

Ad

KRAS ATM EGFR

p.Gly12Val p.Ile2629fs p.Leu858Arg

chr12:25398284 chr11:108203577 chr7:55259514

C CTTATA T

A C G

35%

34%

14%

7

8

Ad Ad

EGFR ATM

p.Leu858Arg p.Ile2629fs

chr7:55259515 chr11:108203577

T CTTATA

G C

32%

30%

TP53 KEAP1

EGFR TP53

p.Ile156Thr p.Arg234Trp

p.Glu746_Ala750del p.Arg241_Arg243del

chr17:7578265 chr19:10602878

chr7:55242465 chr17:7577091

A G

AGGAATTAAGA GAAGC GCCGGTCTCT

G A

A G

63%

40%

38%

70%

9

Ad

10

Ad

EGFR EGFR U2AF1

p.Gly719Cys p.Ser768Ile p.Ser34Phe

chr7:55241706 chr7:55249005 chr21:44524456

G G G

T T A

52%

41%

51%

GCAACATCTCC

11

Ad

EGFR

p.A750-I759 delins GG

chr7:55242478

GAAAGCCAAC GGAGGC AAGGAAATC

44%

AKT3

p.Leu359Val

chr1:243716119

A

C

25%

12

13

Ad Ad

EGFR

-

p.Leu858Arg

-

chr7:55259514

-

T

G

50%

14

Ad

KRAS NFE2L2 CTNNB1

p.Gly12Cys p.Glu82Gln p.Leu427Phe

chr12:25398285 chr2:178098801 chr3:41275113

C C C

A G T

50%

32%

72%

TP53 KMT2D AKT3 SETD2 PTEN

p.Gly115Val p.Pro3145Ser p.Trp410Cys p.Gln97Ter p.Tyr155fs

chr17:7578469 chr12:49431706 chr1:243708833 chr3:47165837 chr10:89692977

C G C G TC

A A A A T

69%

27%

26%

22%

26%

15

Ad

16

Ad

MGA NOTCH2

p.Ser1854Cys p.Gly751Val

chr15:42041366 chr1:120496279

C C

G A

20%

82%

17

Ad

KEAP1

p.Gly417Trp

chr19:10602329

C

A

68%

Ad-(i)

KRAS p.Gly12Ala chr12:25398284 C G 58%

18

TP53 MAP2K1 FOXP2 KRAS

p.Cys238Phe p.Lys57Asn p.Gln250Lys p.Gly12Val

chr17:7577108 chr15:66727455 chr7:114271658 chr12:25398284

C G C C

A T A A

51%

39%

31%

97%

Ad-(ii)

19

Ad

TP53 TP53

p.Val118Phe p.Gly205Cys

chr17:7578461 chr17:7577551

C C

A A

86%

90%

20

Sq

RB1 KMT2D KMT2D

p.Glu112fs p.Val1340fs Splice site

chr13:48916802 chr12:49442891 chr12:49427849

A C C

AC CAG G

43%

37%

36%

SETD2 TP53 PIK3CA MGA CREBBP KMT2D RB1

p.Glu902Gly

chr3:47163421 chr17:7578394 chr3:178936091 chr15:42019515 chr16:3819291 chr12:49444834 chr13:48881510

T T G C C C TG

C C A A A A T

61%

53%

41%

37%

37%

35%

90%

p.His140Arg p.Glu545Lys p.Gln1190Lys p.Glu982Ter p.Glu878Ter p.Glu79fs

21

Sq

22

Sq

TP53 PTEN SMAD4 TP53

p.Met207Val p.Leu140fs p.Arg361His p.Pro152fs

chr17:7577545 chr10:89692935 chr18:48591919 chr17:7578475

T TA G G

C T A GT

82%

79%

72%

85%

23

Sq

NF1 NRAS ARID2 NFE2L2 EP300 KMT2D

p.Tyr2476Phe p.Gln61Lys p.Thr219Ser p.Asp178His p.Ser2328fs p.Arg2830Ter

chr17:29677306 chr1:115256528 chr12:46215221 chr2:178097182 chr22:41574692 chr12:49432651

A G C C

GTCCT G

T T G G

GTCCCT A

50%

49%

45%

44%

41%

29%

Sq-(i)

24

SMARCA4 TP53 FOXP2 RIT1

MGA MGA RB1

p.Glu371Ter p.Arg248Trp p.Pro277Leu p.Thr70Ser p.Asp339His p.Glu1249Lys p.Asn290fs

chr19:11098593 chr17:7577538 chr7:114271740 chr1:155880247 chr15:41962107 chr15:42021449 chr13:48939032

G G C T G G GA

T A T A C A G

75%

74%

64%

43%

39%

35%

35%

Sq-(ii)

25

Sq

TP53 EP300 NOTCH2

splicesite_3 p.Ala2076Ser p.Lys1514Glu

chr17:7578368 chr22:41573941 chr1:120466579

C G T

T T C

74%

57%

39%

26

Sq

TP53 EGFR HRAS EP300 COBL ATM EP300 KMT2D MET

-

p.His154Arg (193) p.Asn476Ser p.Gly12Cys p.Gln259Glu p.Gly1248Asp p.Phe239Ser p.Pro306Ser p.Gly3157Arg p.Glu312Gly

-

chr17:7578271 chr7:55227960 chr11:534289 chr22:41521913 chr7:51092831 chr11:108115568 chr22:41523500 chr12:49431670 chr7:116340073

-

T A C C C T C C A

C G A G T C T T G

85%

75%

59%

48%

34%

25%

25%

22%

21%

27

Sq

28

Sq

KEAP1 RB1 MGA SETD2 KMT2D TP63 NOTCH1 TP53

p.Arg380Gly p.Glu54Ter p.Val1621Leu p.Gly1317Val p.Glu1391Val p.Arg647Leu p.Val868Gly p.Glu300Ter

chr19:10602440 chr13:48881438 chr15:42035019 chr3:47162176 chr12:49441812 chr3:189612188 chr9:139405241 chr17:7574012

T G G C T G GA C

C T C A A T TC A

94%

89%

88%

79%

32%

25%

22%

89%

29

Small

30

LCNEC

NOTCH1 KMT2D PTEN

p.Cys111Ser Splice site p.Ala328Val

chr9:139418240 chr12:49428720 chr10:89720832

C T C

G A T

69%

47%

25%
