# Supplementary material for: Distribution of circulating tumor DNA in lung cancer: analysis of the primary lung and bone marrow along with the pulmonary venous and peripheral blood
Source: Oncotarget. 2017 Jul 25;8(35):59268–81. doi: 10.18632/oncotarget.19538 (PMC5601731; doi:10.18632/oncotarget.19538)
Supplement: Supplementary file 4 [file oncotarget-08-59268-s004.docx]

Supplementary Table 3: The number of cell-free somatic mutations containing ctDNA in analyzed samples

Case

Tumor

Pul.V plasma

Peri.B plasma

BM sup

Pul.V Buffy

BM ppt

1

9

5

16

2

5

2

2

0

0

5

0

1

2

3

11

4

28

2

2

3

4

3

0

2

1

0

1

5

16

3

8

1

4

6

6

12

0

3

6

1

3

7

2

0

0

0

0

0

8

8

0

4

2

3

2

9

5

1

1

0

0

1

10

11

2

11

2

2

3

11

6

0

1

0

0

2

12

3

0

0

1

0

1

13

1

0

0

1

0

0

14

9

3

3

2

1

1

15

13

3

2

3

2

0

16

13

7

5

4

6

1

17

11

1

8

2

1

2

18-(i)

9

1

2

3

3

8

18-(ii)

12

19

9

4

4

2

1

2

20

12

0

3

2

2

2

21

8

3

1

0

0

1

22

8

0

19

0

1

1

23

6

0

2

0

1

0

24-(i)

6

0

0

0

0

0

24-(ii)

7

25

1

0

0

0

0

0

26

4

1

4

0

0

0

27

112

8

6

2

1

1

28

2

1

2

0

1

0

29

9

0

1

1

0

0

30

14

1

6

2

1

0

Total

352

48

147

41

39

45
