# Supplementary material for: Distribution of circulating tumor DNA in lung cancer: analysis of the primary lung and bone marrow along with the pulmonary venous and peripheral blood
Source: Oncotarget. 2017 Jul 25;8(35):59268–81. doi: 10.18632/oncotarget.19538 (PMC5601731; doi:10.18632/oncotarget.19538)
Supplement: Supplementary file 5 [file oncotarget-08-59268-s005.docx]

Supplementary Table 4: The genes targeted in the cancer

| **No** | **Gene symbol** | **Chromosome** | **Number of amplicons** | **Total bases** | **Covered bases** | **Overall coverage** |
| --- | --- | --- | --- | --- | --- | --- |
| 1 | AKT1 | chr14 | 26 | 1573 | 1497 | 95% |
| 2 | AKT2 | chr19 | 27 | 1576 | 1543 | 98% |
| 3 | AKT3 | chr1 | 30 | 1624 | 1624 | 100% |
| 4 | ARID1A | chr1 | 76 | 7058 | 6023 | 85% |
| 5 | ARID1B | chr6 | 75 | 6950 | 5965 | 86% |
| 6 | ARID2 | chr12 | 71 | 5718 | 5643 | 99% |
| 7 | ASCL4 | chr12 | 5 | 532 | 382 | 72% |
| 8 | ATM | chr11 | 147 | 9791 | 9439 | 96% |
| 9 | BRAF | chr7 | 37 | 2481 | 2224 | 90% |
| 10 | CDKN2A | chr9 | 9 | 962 | 612 | 64% |
| 11 | COBL | chr7 | 48 | 4151 | 3977 | 96% |
| 12 | CREBBP | chr16 | 96 | 7639 | 7071 | 93% |
| 13 | CTNNB1 | chr3 | 32 | 2486 | 2486 | 100% |
| 14 | CUL3 | chr2 | 42 | 2561 | 2495 | 97% |
| 15 | EGFR | chr7 | 60 | 4189 | 4135 | 99% |
| 16 | EP300 | chr22 | 90 | 7555 | 7182 | 95% |
| 17 | EPHA7 | chr6 | 44 | 3175 | 3154 | 99% |
| 18 | ERBB2 | chr17 | 57 | 4080 | 3808 | 93% |
| 19 | ERBB3 | chr12 | 59 | 4440 | 4374 | 99% |
| 20 | FGFR1 | chr8 | 41 | 2825 | 2816 | 100% |
| 21 | FGFR2 | chr10 | 43 | 2910 | 2842 | 98% |
| 22 | FGFR3 | chr4 | 34 | 2752 | 2215 | 81% |
| 23 | FOXP2 | chr7 | 36 | 2487 | 2469 | 99% |
| 24 | HRAS | chr11 | 11 | 683 | 683 | 100% |
| 25 | KEAP1 | chr19 | 24 | 1925 | 1845 | 96% |
| 26 | KMT2D | chr12 | 192 | 17154 | 15854 | 92% |
| 27 | KRAS | chr12 | 10 | 737 | 681 | 92% |
| 28 | MAP2K1 | chr15 | 18 | 1292 | 1239 | 96% |
| 29 | MET | chr7 | 59 | 4427 | 4396 | 99% |
| 30 | MGA | chr15 | 110 | 9428 | 9345 | 99% |
| 31 | MLL | chr11 | 144 | 12279 | 11875 | 97% |
| 32 | NF1 | chr17 | 136 | 9161 | 9023 | 99% |
| 33 | NFE2L2 | chr2 | 23 | 1868 | 1826 | 98% |
| 34 | NOTCH1 | chr9 | 99 | 8008 | 7078 | 88% |
| 35 | NOTCH2 | chr1 | 101 | 7809 | 7539 | 97% |
| 36 | NRAS | chr1 | 9 | 610 | 610 | 100% |
| 37 | PIK3CA | chr3 | 50 | 3407 | 3282 | 96% |
| 38 | PTEN | chr10 | 18 | 1302 | 1223 | 94% |
| 39 | RASA1 | chr5 | 55 | 3412 | 3216 | 94% |
| 40 | RB1 | chr13 | 55 | 3057 | 2902 | 95% |
| 41 | RBM10 | chrX | 48 | 3228 | 3079 | 95% |
| 42 | RIT1 | chr1 | 13 | 771 | 771 | 100% |
| 43 | SETD2 | chr3 | 91 | 7905 | 7663 | 97% |
| 44 | SLIT2 | chr4 | 76 | 4972 | 4854 | 98% |
| 45 | SMAD4 | chr18 | 24 | 1769 | 1715 | 97% |
| 46 | SMARCA4 | chr19 | 74 | 5399 | 5055 | 94% |
| 47 | SOX2 | chr3 | 9 | 964 | 883 | 92% |
| 48 | STK11 | chr19 | 23 | 1392 | 1343 | 97% |
| 49 | TP53 | chr17 | 22 | 1383 | 1351 | 98% |
| 50 | TP63 | chr3 | 34 | 2360 | 2227 | 94% |
| 51 | TSC1 | chr9 | 49 | 3705 | 3603 | 97% |
| 52 | TSC2 | chr16 | 92 | 5834 | 5677 | 97% |
| 53 | U2AF1 | chr21 | 15 | 880 | 870 | 99% |
